# Suprachiasmatic Nuclei Possess Glucocorticoid Receptors That Activate Downstream Signaling Pathways but Do Not Entrain Their Circadian Clock

**DOI:** 10.1111/apha.70138

**Published:** 2025-11-28

**Authors:** Martin Sládek, Vendula Lužná, Pavel Houdek, Alena Sumová

**Affiliations:** ^1^ Laboratory of Biological Rhythms Institute of Physiology of the Czech Academy of Sciences Prague Czech Republic

**Keywords:** circadian clock, entrainment, fetus, glucocorticoids, ontogenesis, suprachiasmatic nuclei

## Abstract

**Aim:**

The circadian clock in the suprachiasmatic nuclei of the hypothalamus (SCN) is resistant to glucocorticoids (GC) in adults but responds to dexamethasone (DEX) during the fetal stage. Previously, this resistance of the adult SCN clock was attributed to a developmental loss of the glucocorticoid receptor (GR). The aim of our study was to re‐examine the mechanism underlying SCN clock resistance.

**Methods:**

We detected GR in the adult SCN at the mRNA level (*Nr3c1*) using RT‐qPCR and at the protein level by immunohistochemistry, and examined the effects of DEX on the SCN clock of *mPer2* ^*Luc*^ mice ex vivo at embryonic day E17, postnatal days P1–2, P3, P5, P10, and adulthood.

**Results:**

Surprisingly, we found that *Nr3c1* expression gradually increases from the fetal stage to postnatal day (P)28. In the adult SCN, GR immunoreactivity is present in both neurons and glia. The effect of DEX on the SCN clock disappears shortly after birth. Although DEX does not entrain the adult SCN clock, it acutely increases the expression of *Gilz* and *Sgk1*, indicating that GRs in the adult SCN can activate downstream signaling pathways. Inhibition of glial metabolism by fluorocitrate had no effect on resistance to DEX, but treatment with tetrodotoxin sensitized the clock to DEX and induced phase shifts similar to those observed at the fetal stage.

**Conclusion:**

These results indicate that the adult SCN possesses GRs capable of activating GC‐signaling pathways, but the clock is resistant to GC in part due to coupling between individual cellular oscillators.

## 1 Introduction

The release of glucocorticoids (GC) from adrenal glands is precisely controlled by the circadian system [1] to align it with the actual arousal state; their levels elevate preceding awakening time and reach nadir during sleep time [2]. In addition, they are acutely elevated any time of day upon exposure to stressful situations (reviewed in [3]). The circadian system controls both the hypothalamic–pituitary–adrenal (HPA) axis [4, 5] and the GC daily rhythm via a complex mechanism that requires communication between the central clock in the suprachiasmatic nuclei of the hypothalamus (SCN) and the peripheral clock in the adrenal glands [6, 7, 8]. The relationship between GCs and the circadian system is reciprocal—the system drives the rhythm in their levels and the rhythm provides the system with the signal to synchronize cellular clocks in various parts of the body [9, 10]. In addition, the release of GCs follows a distinct ultradian rhythm (reviewed in [11]), which persists in the absence of the SCN [12]. The canonical molecular mechanism by which glucocorticoids (GCs) influence the circadian clock involves the transactivation of GC‐response elements (GREs) in the promoters of core clock genes such as *Per1*, *Per2*, and *Nr1d1* [13, 14]. This affects the levels of clock gene transcripts and their protein products, thereby influencing the stoichiometry of the transcriptional‐translational feedback loops that generate the circadian signal, ultimately leading to entrainment of the clocks [15]. There are additional genomic (transrepression of transcription factors such as AP‐1) and non‐genomic mechanisms (rapid effects via membrane‐bound GRs interacting with various kinase cascades) by which glucocorticoids can influence cellular processes [16, 17, 18, 19]. Due to the ubiquitous presence of GR and the precise control of GC levels by the circadian system, these hormones spread information about the actual time of day to cellular clocks in all tissues throughout the body (reviewed in [20]), synchronizing the clocks in peripheral organs [16, 21] as well as in the brain [19, 22]. The remarkable exception is the central clock in the SCN, which is greatly resilient to the GC signal [9, 23, 24, 25]. This feature is considered a functional hallmark of the central clock, which entrains to regularly repeating environmental cycles, but some degree of stability and resilience to abruptly elevated signals upon unpredictable stimuli is required. For decades, the mechanism behind this feature has been explained by the absence of glucocorticoid receptors (GR) in the SCN, based on a study in which GR‐immunoreactivity was present during early developmental stages but not detected in the SCN of adult rats [23]. These data suggested that the SCN resilience to GCs is specific to the adult stage.

We recently found that resistance to GCs is absent during the perinatal stage, as the synthetic GC analog, dexamethasone (DEX), efficiently reset the clock in ex vivo hypothalamic explants containing fetal SCN [26]. In addition, the stress‐induced increase in endogenous GC levels shifted the rhythm of *Bmal1* expression in the SCN of pups shortly after birth [27]. In both studies, the effects were blocked by the GR antagonist mifepristone. In this study we investigated the developmental acquisition of insensitivity to DEX in the SCN. We tracked this process from embryonic through postnatal stages to adulthood using in vivo experiments in rats and in vitro studies with organotypic SCN explants from *mPer2* ^*Luc*^ mice. We also investigated the presence, localization and ability to activate downstream pathways of the GR in the adult SCN in vivo and tested the role of inter‐neuronal communication in the mechanism of resilience of the SCN clock to DEX. Our data challenge the current view on the mechanism of SCN resistance to GCs.

## 2 Materials and Methods

### 2.1 Animals

Experiments were conducted with adult male and female Wistar:Han rats (Institute of Physiology of the Czech Academy of Sciences) and *mPer2* ^*Luc*^ mice [28]. Animals were kept under a regime with 12 h of light and 12 h of darkness (LD12:12, lights on at 06:00 a.m., designated Zeitgeber time 0) at 21°C ± 2°C with free access to food (standard diet) and water. Female rats or mice were mated with males, and those with positive sperm in vaginal smears (assigned as embryonic day E0) were housed individually. The dams and their pups were maintained undisturbed under LD12:12 until weaning. Adult rats and pups from 3 days of age were sacrificed under deep anesthesia by injections of a mixture of ketamine (Vétoquinol, s.r.o., Czech Republic; 120 mg/kg) and xylazine (Bioveta a.s., Czech Republic; 12 mg/kg). Mice of all ages and rat pups up to 1–2 days of age were sacrificed via rapid cervical dislocation.

### 2.2 Animal Protocols

To detect the effect of DEX on the PER2‐driven bioluminescence in the SCN of *mPer2* ^*Luc*^ mice ex vivo, brains were collected from fetuses at embryonic day 17 (E17), from pups at postnatal days (P)1/2, P3, P5, P10, and from adults (mean 6‐month‐old ±2 months). SCN explants were prepared and treated for bioluminescence monitoring as described below.

For the detection of 24 h gene expression profiles, 5 rats were sacrificed every 4 h during the 24‐h cycle to collect the brains of pups on postnatal days (P)2, P10, P20, and P28. To collect brains from the fetuses at E19, 5 fetuses from one pregnant rat were sacrificed at each time point.

To demonstrate the acute effects of dexamethasone (DEX) in vivo, adult male rats were injected with 0.5 mL dexamethasone (DEX, 1 mg/kg i.p.) or vehicle (phosphate‐buffered saline) at 9 a.m. (ZT3) and sacrificed 1, 2, and 4 h after the injection to collect brains (*n* = 4–5/time point). The dose of DEX was selected based on previous studies [19], and the timing of injection was chosen to coincide with low levels of endogenous GCs in rats [29]. Whole brains were immediately frozen in dry ice and stored at −80°C. For RT qPCR, brains were cryosectioned into 20‐μm thick coronal sections containing the medial part of the rostro‐caudal extent of the SCN as visualized with cresyl violet staining (Sigma‐Aldrich, St. Louis, MI, USA). The SCN was precisely separated bilaterally using a laser microdissector (LMD6000, Leica), as we have previously described elsewhere [30].

### 2.3 Bioluminescence Monitoring In Vitro

The brains of E17‐P3 mice were cut into 300‐μm coronal sections using a tissue chopper, while brains from P5, P10 and adult mice were immediately sectioned into 250‐μm coronal sections in cold Earle's Balanced Salt Solution (EBSS, Sigma) using a vibratome (Leica, Germany). The slice containing the medial SCN region was dissected and immediately placed on Millicell Culture Inserts (Merck) inside 35‐mm Petri dishes containing 1 mL of recording medium. Explants were kept in air‐buffered recording media containing DMEM (Sigma) supplemented with 2% B27 supplement (Thermo Fisher), 0.1 mM D‐Luciferin (Biosynth, Switzerland), 100 U/mL penicillin, 100 μg/mL streptomycin and 2 mM GlutaMAX (Thermo Fisher). The explants were placed in the LumiCycle apparatus (Actimetrics, Wilmette, IL, USA) to monitor bioluminescence at the tissue levels. Explants were treated with either 100 nM DEX (1 μL of 0.1 mM DEX in 1% ethanol/1 mL of recording medium) or vehicle (1% ethanol) 3–5 days after the addition of fresh recording medium. Some explants were treated repeatedly after washout and at least 7 days of resting to reduce the number of sacrificed animals. To inhibit glia, DL‐fluorocitrate barium salt (FLC, Sigma) was used according to Paulsen, Contestabile et al. [31] to prepare a fresh 7 mM FLC stock; 7 μL of FLC or VEH (identically prepared stock without FLC) was added to 1 mL of recording medium 48 h before the treatment with DEX. To block the Na^+^ channels, 1 μM tetrodotoxin (Sigma) was present in the recording medium before treatment with DEX.

To image the PER2 luminescence signal at the microscopic level, 14 explants were placed in the motorized luminescence microscope Luminoview LV200 (Olympus, Japan) with LUCPLFLN40X objective (Olympus) and the EMCCD camera ImageEM X2 (Hamamatsu, Japan) water cooled by Minichiller 280 (Huber, Germany); luminescence signal was imaged every hour with an exposure time of 10 min and 250 electromagnetic gain. One micromole of tetrodotoxin (Sigma) was present in the recording medium to block the Na^+^ channels; to avoid affecting the position of the explant above the objective, instead of adding DEX (*n* = 7) or VEH (*n* = 7) to the medium after 3 days of imaging, 5 μL of pre‐diluted DEX (200 nM) or VEH was dropped directly on top of the explant.

### 2.4 Luminescence Data Analysis

Lumicycle Analysis (Actimetrics, USA) was used to analyze the raw bioluminescence data. Data were baseline‐subtracted with the running 24‐h average prior to fitting to a sine wave to calculate the amplitude, period and phase shift before and after treatments. Treatment‐induced phase shifts in the bioluminescence rhythms were quantified by fitting a sine curve to the first at least 3 complete circadian cycles of a 24‐h running average baseline‐subtracted rhythm and then extrapolating beyond the time of the treatment (reflecting the original phase). The resulting absolute phase shift was calculated as a difference between the extrapolated sine curve and the post‐treatment luminescence trace (reflecting the new phase). The shift was assigned as a phase advance (+) or a phase delay (−). The phase response curve (PRC) was constructed by plotting the calculated phase shift as a function of the time of the treatment normalized to the endogenous period in vitro and expressed relative to the trough (treatment time, tt0) or peak (tt12) of the rhythm. For statistical comparisons between DEX and VEH PRCs, the data for the phase shifts between tt0 and tt13.3, and tt13.3 and tt24 were fitted with linear regression curves and their slopes were compared by *F*‐test. The phase transition curve (PTC) was constructed by plotting the peak of the first full cycle after the treatment (*y*, new phase) as a function of the peak of the extrapolated sine curve (*x*, old phase). The PTC data were plotted as a tetraplot for clarity. CellSens Dimensions (Olympus) was used to acquire LV200 images and export them as 16‐bit TIFFs. Cosmic rays were removed by pixel‐wise subtraction of consecutive TIFFs. Whole SCN were manually traced in Fiji ImageJ and algorithmically subdivided into approximately cell‐sized regions of interest (ROIs, on average cca. 500/explant). Data were exported as mean intensity signal and *xy* coordinates were analyzed after detrending and denoising using a custom modified Python script based on the per2py package [32].

### 2.5 Immunohistochemistry

The adult mice and rats were deeply anesthetized with a mixture of ketamine (Vétoquinol, s.r.o., Czech Republic; 120 mg/kg) and xylazine (Bioveta a.s., Czech Republic; 12 mg/kg) and transcardially perfused with phosphate buffer (PBS) followed by paraformaldehyde (4% in PBS, Sigma). Brains were removed and postfixed in 4% PFA for 12 h at 4°C and cryoprotected in 20% sucrose‐PBS overnight at 4°C. Coronal 30‐μm‐thick sections were cut with a freezing microtome (CM1850, Leica, Germany) and free‐floating sections were processed for immunofluorescence methods as previously described [33, 34]. After the standard antigen retrieval procedure in citrate buffer (0.3% in PBS), the sections were incubated in 2% normal goat serum (abcam ab7481) for 60 min. The primary polyclonal antibodies applied overnight at 4°C were as follows: GR (M‐20) 1:100, rabbit, Santa Cruz sc‐1004; FOX2 1:500, mouse, abcam ab57154; GFAP 1:1000, chicken, abcam ab4674; ELAVL4 1:600, mouse, Millipore MABN153. The secondary antibodies (incubation for 1 h) were goat anti‐rabbit, 1:1000, Alexa Fluor 594, Thermo Fisher A‐11037; goat anti‐mouse, 1:1000, Alexa Fluor 488, Thermo Fisher A‐11001; goat anti‐chicken, 1:1000, Cy3, Abcam ab97145. The images were taken with the Leica SP8 WLL MP laser scanning confocal microscope.

### 2.6 RT qPCR

Dissected rat SCN tissues were collected in a microfuge tube containing RLT buffer from the RNeasy Micro kit (Qiagen, Valencia, USA) and stored until RNA isolation. RNA was isolated using the RNeasy Micro kit (Qiagen); the whole elution volume was then reverse‐transcribed using a High Capacity cDNA RT Kit (ThermoFisher). Diluted cDNA was then amplified on LightCycler480 (Roche) using SYBR Select qPCR Master Mix (ThermoFisher) as described previously [30]. Reverse transcription‐quantitative polymerase chain reaction (RT qPCR) method was used to detect *Nr3c1* (Forward: CAGAGAATGTCTCTACCCTG, Reverse: CTTAGGAACTGAGGAGAGAAG) mRNA levels in the SCN (3–5 replicates/time point). Relative quantity was calculated using Livak's ΔΔCt method against the reference gene *Tbp* (for 24 h expression profiles, For: CATCATGAGAATAAGAGAGCC, Rev.: GGATTGTTCTTCACTCTTGG) or *B2m* (for the acute effect of DEX, For: CGCTCGGTGACCGTGATCTTTCTG, Rev.: CTGAGGTGGGTGGAACTGAGACACG). All samples were analyzed in the same RT qPCR run. The reference gene *Tbp* was used for 24 h expression profiles designed to detect developmental changes in the SCN, as its expression was stable during development. The reference gene *B2m* was used to detect the acute effects of DEX in the SCN of adult animals.

### 2.7 Statistics

**FIGURE 1:**
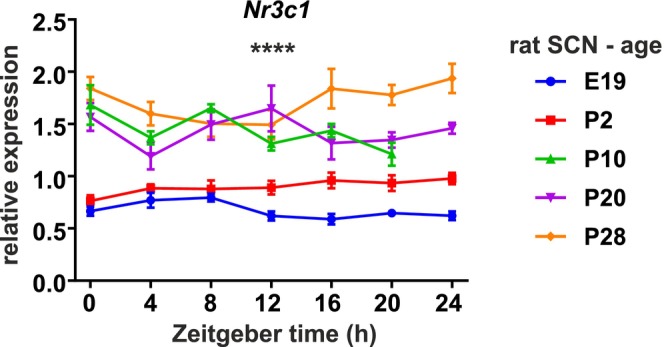
Expression of glucocorticoid receptor gene *Nr3c1* in the rat SCN increases during the development. Total RNA was isolated every 4 h at embryonic age E19 (blue) and postnatal ages P2 (red), P10 (green), P20 (purple) and P28 (orange) from rats kept on light: Dark cycle LD12:12. Two‐way ANOVA shows significant effect of age (****p < 0.0001) between the expression at E19 and P28.

**FIGURE 2:**
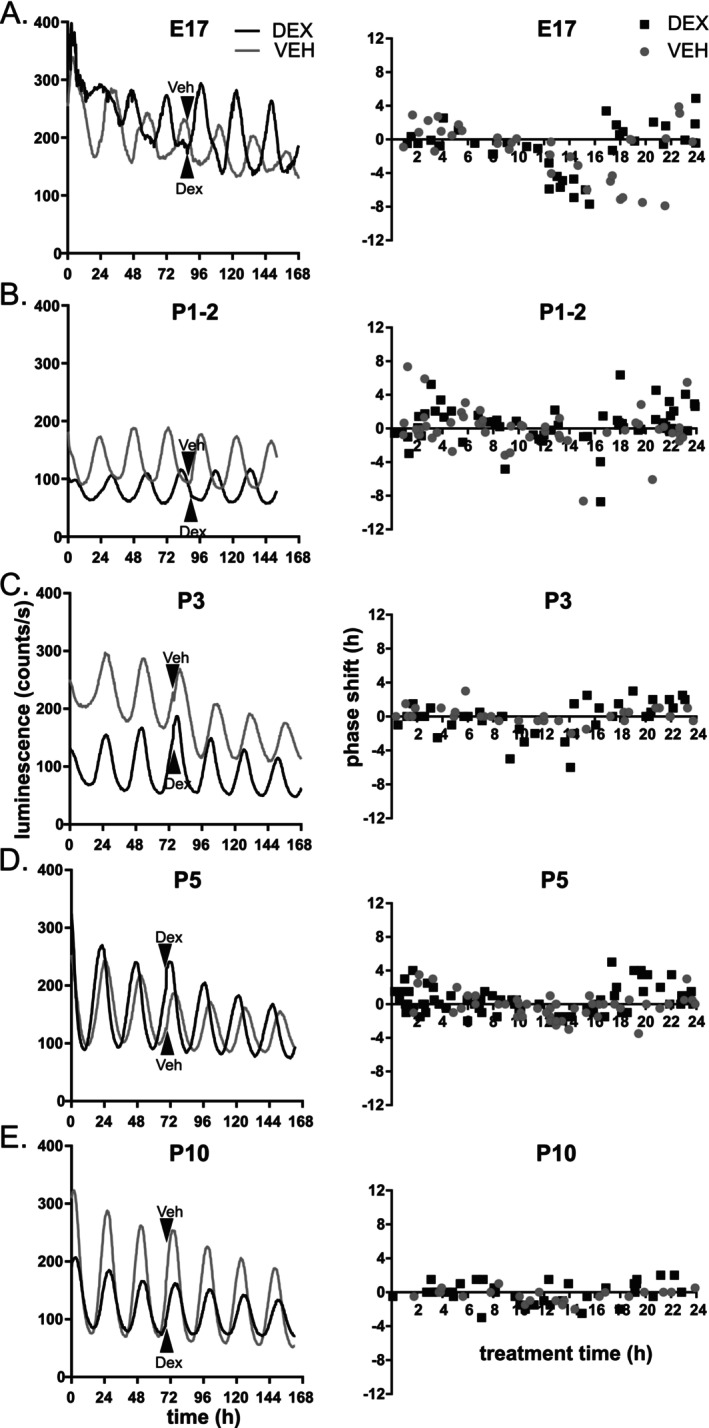
The sensitivity of the explanted mouse SCN to dexamethasone (DEX) and a general effect of manipulation with the explant (VEH) decreases during ontogenetic development. (A) Representative PER2 luminescence traces (left) and the full phase response curve (PRC) to DEX (100 nM) and VEH treatment (right) in the SCN explanted from embryonic day 17 (E17) fetuses. (B–E) Traces and PRCs of SCN explanted from postnatal ages (P) 1–10. PRC plots the circadian time (CT) of the treatment with VEH (gray circles) or DEX (black squares) (with CT0 set to the trough and CT12 to the peak of the PER2 luminescence) on *x*‐axis, and resulting phase advances (positive) or delays (negative) in hours on *y*‐axis. Arrows mark the time of DEX or VEH application to the medium.

RT qPCR profiles of *Nr3c1* gene expression were analyzed by two‐way ANOVA with Šidák's multiple comparisons test to compare E19 and P28 samples (Figure 1). Period and amplitude of PER2 luminescence in adult SCN explants were compared by the Mann–Whitney test (Figure 2D–F). SCN genes in rats injected with DEX or VEH were measured by RT qPCR and analyzed by two‐way ANOVA with Šidák's multiple comparisons test (Figure 4). Amplitude of SCN explants treated with FLC/TTX and then with either DEX or VEH was compared before and after the treatment by Wilcoxon matched‐pair test (Figure 6B,C,G), while the partial PRCs were fitted with linear regression and the slopes were compared by *F*‐test (Figure 6H). Immediate amplitude increase in individual cell‐sized rois measured in 9 DEX‐ and 9 VEH‐and‐TTX‐treated explants was analyzed by the Mann–Whitney test (Figure 6J). *p* < 0.05 was required to report significance. Statistical tests implemented in Prism 7 (Graphpad, USA) and SciPy stats Python module were used.

## 3 Results

### 3.1 Nr3c1 Expression in the SCN Increases With Age

We used RT qPCR to analyze *Nr3c1* expression profiles in microdissected SCN samples during the developmental stages of E19, P2, P10, P20, and P28 (after the end of weaning) (Figure 1). Surprisingly, we found a gradual increase in *Nr3c1* expression during ontogenesis, with the lowest mRNA levels of the GR gene at E19 and the highest levels at P28 (two‐way ANOVA comparison of E19 vs. P28, age effect *p* < 0.0001, time effect *p* = 0.4, interaction effect *p* = 0.03).

### 3.2 Developmental Loss of the Response to DEX

Since we found that GR levels in the SCN increase rather than decrease during development (as previously reported), we investigated the responses of the circadian clock in the SCN to the glucocorticoid analog dexamethasone (DEX, 100 nM) during five distinct perinatal developmental stages and in adulthood. To this end, we constructed phase response curves (PRCs) by analyzing the phase shifts induced by DEX treatments of SCN explants from E17, P1–2, P3, P5 to P10 at various time points to cover a 24‐h interval relative to the peak/trough of the PER2‐bioluminescence rhythm (Figure 2). At E17 (Figure 2A), the SCN clock was sensitive to the manipulation itself (VEH treatment), but DEX induced phase advances at the time of PER2‐bioluminescence decline, whereas VEH induced significant phase delays (tt16‐20). The sensitivity window was consistent with the results of our previous study in which we used a different method to prepare the SCN explants [26]. The PRC for P1‐2 explants showed responses to both DEX and VEH that were randomly spread over a 24‐h interval (Figure 2B). The magnitudes of phase shifts induced by both VEH and DEX progressively decreased at postnatal days (P)3 and P5 (Figure 2C,D), with little to no detectable phase shift until P10 (Figure 2E). The results showed that the SCN clock gradually gains resistance to DEX during the early postnatal period.

**FIGURE 3:**
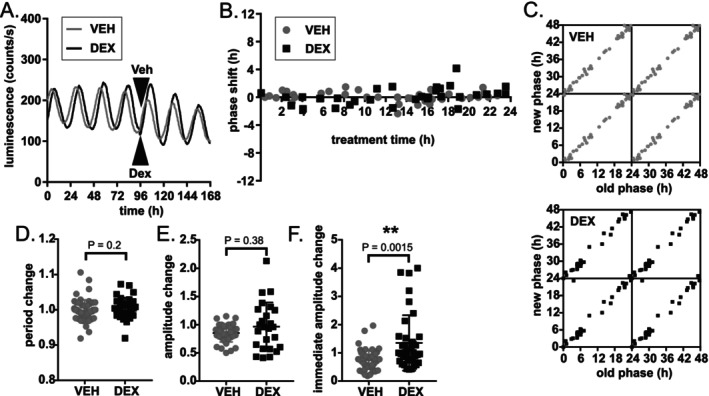
DEX does not reset the clock in the adult mouse SCN. (A) Representative PER2 luminescence traces of SCN treated with VEH (gray) or DEX (black), arrows mark the time of treatment. (B) PRC to DEX (black squares) or VEH (gray circles). (C) Phase transition curves (PTCs) to DEX (black squares) or VEH (gray circles); tetra‐plotted data show original (old) phase on *x*‐axis and the resulting (new) phase after the treatment on *y*‐axis. (D) Relative period (period measured 3 cycles after the treatment divided by period measured 3 cycles before the treatment) and relative amplitude (E) was not different between DEX and VEH‐treated explants. (F) DEX treatment resulted in a significant temporary increase in amplitude (measured over one circadian cycle after/before the treatment). Data analyzed by Mann–Whitney tests. ** *P* < 0.01.

Next, we focused in more detail on the responses of the adult SCN clock to DEX. Construction of the full PRC confirmed that DEX did not induce significant phase shifts in the adult SCN clock (Figure 3A–C). There were also no significant persistent effects on period (Figure 3D) or amplitude (Figure 3E) measured over 3 days post‐treatment. Interestingly, DEX caused a rapid temporary increase in amplitude within 24 h after DEX treatment (Figure 3F).

### 3.3 GR in the Adult SCN Is Able to Activate Downstream Signaling Pathways

**FIGURE 4:**
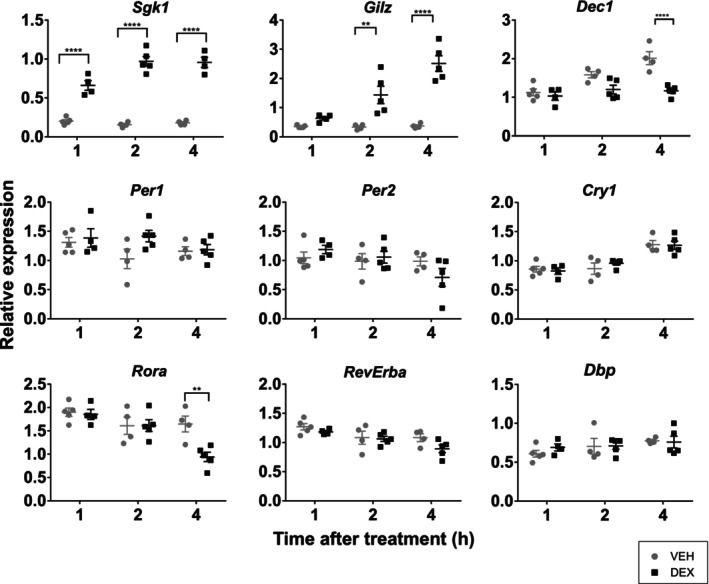
Glucocorticoid receptor in the adult SCN is able to activate downstream signaling pathways. Adult rats were injected with DEX (black squares) or VEH (gray circles) and sacrificed 1, 2, or 4 h after; expression of GR activation markers (*Sgk1*, *Gilz*) and clock genes (*Dec1*/*Bhlhe40*, *Per1*, *Per2*, *Cry1*, *Rora*, *Rev‐Erba*/*Nr1d1*, *Dbp*) was then measured in the SCN by RT‐qPCR and analyzed by two‐way ANOVA with Šidák's post hoc test, **p < 0.01, ****p < 0.0001.

The finding of acute DEX‐induced increase in the amplitude of the PER2‐bioluminescence rhythm in the adult SCN prompted us to test whether GR can activate canonical GC signaling pathways or affect clock gene expression in vivo. We injected adult rats with either DEX (1 mg/kg i.p.) or VEH at ZT3 and detected the expression of selected genes in microdissected SCN samples 1, 2, or 4 h later (Figure 4). We found a highly significant increase in the expression of Serum and glucocorticoid‐regulated kinase 1 (*Sgk1*, already 1 h after injection, two‐way ANOVA with Šidák's post hoc test, *p* < 0.0001) and Glucocorticoid‐induced leucine zipper protein (*Gilz*/*Tsc22d3*, 2 h after injection, *p* = 0.0015), both targets of GR transactivation. We also analyzed the expression of the clock genes *Dec1*, *Per1*, *Per2*, *Cry1*, *Rora*, *Rev‐ErbA* and *Dbp*. Only *Dec1* (*p* < 0.0001) and *Rora* (*p* = 0.0028) responded to DEX injection after 4 h, while the other clock genes measured were not significantly affected. In contrast to *Sgk1* and *Gilz*, mRNA levels of both *Dec1* and *Rora* decreased by approximately 50% in response to GR activation, indicating a possibility of a noncanonical mechanism of GR action on the SCN clock.

### 3.4 Adult SCN Possess GR in Both Neurons and Glia

**FIGURE 5:**
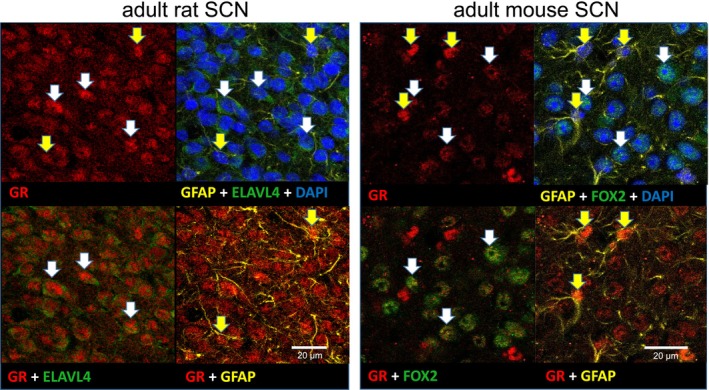
GR protein is detectable in adult rat and mouse SCN in both neurons and glia. Confocal images of immunofluorescence in the adult rat (left) and mouse (right) SCN; red—GR (nuclear signal), yellow—GFAP (cytoplasmic marker of glia), green—either ELAVL4 (rat cytoplasmic marker of neurons) or FOX2 (mouse nuclear marker of neurons), blue—DAPI (DNA marker); yellow arrows highlight glia and white arrows mark neurons with detectable GR.

We used immunofluorescence to spatially and phenotypically identify cells expressing the GR protein in the adult SCN. Since the experiments were performed both in rats and mice, we processed brains from both species. The results showed that GR is expressed in most cells of the adult SCN. We co‐localized GR with neuronal (ELVAL4 in rats, FOX2 in mice) and glial (GFAP in both species) markers. Confocal immunofluorescence images of the adult SCN (Figure 5, left for a rat, right for a mouse) confirmed the presence of GR immunoreactivity in both glia (yellow arrows) and neurons (white arrows).

### 3.5 FLC Does Not Sensitize the Adult SCN to DEX

**FIGURE 6:**
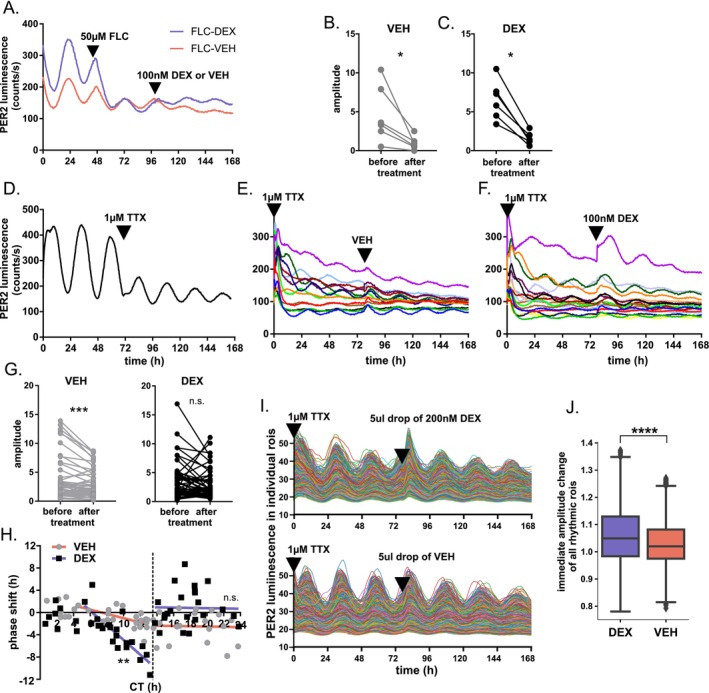
Blocking of sodium channels sensitizes the SCN clock to DEX, while inhibition of glia does not. (A) Representative traces of SCN explants treated with metabolic inhibitor of glia fluorocitrate (FLC, 50 μM), followed by either 100 nM DEX (blue) or VEH (red). FLC‐treated explants were then analyzed before and after the addition of either VEH (B) or DEX (C); amplitude was analyzed by Wilcoxon pair test, *p < 0.05. (D) Representative trace of PER2 luminescence in explanted mouse SCN and the effect of 1 μM TTX on its amplitude. Traces of SCN explants with TTX in the medium and treated with (E) VEH or (F) 100 nM DEX. (G) DEX stops the dampening of the oscillations in TTX‐treated SCN explants. PER2 rhythms from (E, F) were analyzed before and after the addition of either VEH (gray) or DEX (black); amplitude was analyzed by Wilcoxon pair test, ***p < 0.001. (H) PRC for SCN explants treated with TTX and DEX (black squares) or VEH (gray circles). To visualize the effect of increased phase response, results were divide to approximately subjective “day,” “first and second half of the night” datasets and fitted with linear regression. *F*‐test shows significantly larger slope for DEX‐treated (full line) than VEH‐treated explants (dashed line) during the first half of the subjective night, *p* = 0.0003. (I) PER2 luminescence in individual approximate‐cell‐sized regions of interest (rois) in SCN explant treated with TTX and 5‐μL drop of either 200 nM DEX or VEH. (J) Temporary activation of GR by DEX significantly increases amplitude of PER2 luminescence in individual cell‐sized rois, measured over one circadian cycle after/before the treatment and calculated from all rois in 9 DEX‐ and 9 VEH‐treated explants; analyzed by Mann–Whitney test, ****p < 0.0001.

To investigate whether glia play a role in the resistance of the SCN clock to GC, we tested the effect of an inhibitor of glial metabolism (50 μM fluorocitrate, FLC) on the parameters of the circadian clock in the adult SCN of *mPer2* ^*Luc*^ mice. As expected, the impairment of glial metabolism by FLC decreased the amplitude of circadian oscillations compared to VEH (Figure 6A). However, this did not affect the response to DEX, as we found a comparable decrease in amplitude after treatment with VEH (Figure 6B, Wilcoxon paired test, *p* = 0.03) or DEX (Figure 6C, *p* = 0.03). The result suggests that glia do not play a dominant role in the previously observed effect on PER2 amplitude.

### 3.6 Disturbance of Intercellular Communication by TTX Sensitizes Adult SCN to DEX

To reduce the intercellular coupling among individual neuronal oscillators, we treated the adult SCN explants from *mPer2* ^*Luc*^ mice with Na^+^ channel inhibitor (1 μM tetrodotoxin, TTX) (Figure 6D–H). As expected, TTX acutely decreased the amplitude of the overall PER2‐bioluminescence rhythm analyzed in Lumicycle (Figure 6D). On the third day after the TTX application, we treated the explants with vehicle (Figure 6E) or 100 nM DEX (Figure 6F) and analyzed the effects on the amplitude (Figure 6G), measured 3 days before and after the treatment. Due to a progressive dampening of oscillations in the adult SCN with largely electrically uncoupled neurons, we detected a significant difference in amplitude before and after the treatment with VEH (Figure 6G left, Wilcoxon pair test, *p* < 0.0005); however, treatment with DEX prevented the dampening, because there was no significant difference in amplitude before and after the treatment (Figure 6G right, *p* = 0.18). Therefore, we analyzed the phase response to DEX in electrically uncoupled SCN by constructing the full PRC (Figure 6H) for 100 nM DEX (black squares) and VEH (gray circles). Both compounds produced phase shifts with directions and magnitudes dependent on the timing of the treatment. The effect of VEH on the phase of the TTX‐treated SCN suggests weakening of the robustness of the oscillator. We compared the resulting PRCs for the DEX and VEH by fitting regression lines approx. corresponding to the first and second half of the subjective night (determined by the transition points set at tt 4.5 and 13.5 h) (Figure 6H). The statistical comparison of the slopes revealed that in contrast to VEH, the DEX treatments induced significantly larger phase delays (*F*‐test, *p* = 0.0003) but not phase advances (*p* = 0.6) in electrically uncoupled adult SCN.

To reveal the dynamics of the response to DEX in TTX‐treated adult SCN at the single‐cell level, we used luminescence microscopy (Olympus LV200) and analyzed PER2 levels in approximately cell‐sized regions of interest (rois) recorded every 1 h for 72 h before explants were temporarily treated with either 200 nM DEX or VEH (a drop on top of the explant, Figure 6I). While TTX reduced their amplitude, the majority of rois remained significantly rhythmic and largely synchronized with each other. The recording confirmed a transient increase in the amplitude of the rhythm in individual rois in DEX‐ treated but not in VEH‐treated explants (Figure 6J, *n* = 9 explants/group, Mann–Whitney test, *p* < 0.0001). The mild effect is likely due to only a transient activation of GR, because in contrast to previous experiments the drop of 200 nM DEX is rapidly diluted in the medium below the tissue insert (eventually leading to a 200‐fold reduction from the initial concentration).

Altogether, the results show that TTX further increases the sensitivity of the adult SCN to DEX.

## 4 Discussion

The SCN clock is on the top of the hierarchy of the circadian clocks in the body because of its complex structure allowing thousands of cellular oscillators to couple via mutual interconnection to produce a coherent robust signal [35, 36]. Our data suggest that this feature contributes to the mechanism providing the adult SCN with the resilience to GCs, disputing the previous explanation of the mechanism based on the developmental loss of GR. We confirm that the adult SCN contains GR that is able to activate downstream signaling pathways, but their activation does not shift the clock.

The resilience of the adult SCN to GC and stress exposure has been described [37, 38, 39]. Our previous study showed that the fetal SCN clock is sensitive to DEX, because application of DEX to the hypothalamic explants containing the SCN (bulk tissue) induced phase shifts, magnitudes and directions of which were dependent on the time of the application [26]. In addition, application of DEX to pregnant rats affected levels of *c‐fos* mRNA in the laser dissected fetal SCN [26]. In this study, we repeated the experiment, but for better comparison with the data obtained from the SCN explants at later postnatal and adult stages, we prepared coronal slices of the fetal brain containing the SCN of *mPer2* ^*Luc*^ mice. Complete PRC confirmed that the effects of DEX on the fetal SCN differed from that for vehicle mostly at the time of the PER2‐bioluminescence decline, that is, at the same time as in the previous study [26]. During the postnatal development, the sensitivity to DEX gradually disappeared, as demonstrated by PRCs for DEX at P1‐2, P3, P5, and P10.

In the adult SCN explants, DEX did not induce phase shifts that would differ in magnitudes from those induced by the vehicle. Analyses of the developmental changes in *Nr3c1* expression showed that the SCN resistance to DEX was not due to the developmental loss of GR‐immunoreactivity as generally thought based on the study by Rosenfeld and colleagues [23]. Surprisingly, we found the opposite; the expression levels of *Nr3c1* gradually increased in the rat SCN from the fetal stage (E19) through P2, P10, P20 to P28, and exhibited shallow circadian variation in the SCN at E19 and P28. This was unexpected, as other studies using either in situ hybridization [9] or RT‐qPCR [40] failed to detect significant levels of GR mRNA in the mouse SCN. It is possible that it is due to methodological differences. Although we did not perform absolute quantification or comparison with expression in other brain regions, the Ct values for *Nr3c1* were in a similar range to the median Ct values of *Per2* and *Nr1d1*. In addition, we detected GR protein by immunohistochemistry in the adult SCN and the application of DEX to animals at the time of the endogenous trough of GC levels acutely induced the expression of GC‐sensitive genes (*Gilz*, *Sgk1*). Interestingly, we found that DEX decreased the expression of *Dec1* (*Bhlhe40*) in the adult SCN, similarly to what was previously reported in peripheral tissues [41, 42, 43]. *Dec1* is regulated by CLOCK and BMAL1, which bind to E‐box elements in its promoter, but it also responds to various stimuli, including hypoxia [44] and vitamin D3 [45]. DEC1 forms a basic helix–loop–helix transcription factor, which then represses the expression of other clock genes [46]. We may thus speculate that its downregulation by DEX is the mechanism by which GR affects the amplitude of the SCN clock. DEX also significantly decreased the expression of *Rora* (Retinoic acid receptor‐related orphan receptor alpha), an orphan nuclear receptor that activates the transcription of *Bmal1* [47], potentially forming another pathway by which the GR affects the clock [48]. DEX had no effect on the expression of any of the other studied clock genes. This however does not exclude the possibility of an effect via a non‐genomic mechanism [49]. Similarly, we previously reported that a single injection of DEX induced the expression of *Gilz* but not *Per1*, *Per2* and *Nr1d1* in the hippocampus [22], but it was able to induce the expression of both *Gilz*, *Sgk1* and *Per1* in the non‐neuronal extra‐SCN brain clock located in the choroid plexus [19]. Nevertheless, the same injection of DEX in adrenalectomized rats restored the *Per1* expression rhythm in the dentate gyrus of the hippocampus [22]. Other studies found that *Per1* expression is induced in the HPC by exposure of animals to stress [37, 38, 50]. In accordance, a single injection of corticosterone acutely induced a transient burst in *Per1* nascent transcript in this brain region [51] and pulsatile secretion of GC is required for steady‐state *Per1* mRNA expression [11].

The fetal and adult SCN differ mainly in the degree of cellular complexity (neurons and glia) and the level of intercellular communication that is almost lacking in the SCN before birth [52]. In the SCN of adult rats and mice, the GR‐immunoreactivity co‐localized with the neuronal marker as well as the glial marker. The disturbance of glia metabolism by fluorocitrate had no effect on the resistance of the adult SCN to DEX. In contrast, disturbance of inter‐neuronal coupling by TTX, which resulted in an immediate decrease in the amplitude of the bioluminescence rhythm, sensitized the adult SCN clock to DEX applied on the explants on the 3rd day after the TTX treatment. The TTX‐treated SCN exhibited significantly larger phase delays and phase advances of the bioluminescence rhythm after DEX compared to vehicle. In addition, DEX protected against faster dampening of the bioluminescence rhythm induced by TTX. The TTX was shown to weaken coupling between the oscillators [53, 54]. In our setup, the decoupling between individual “cellular‐like” rhythms after the TTX treatment was not as prominent as expected. It was likely because the monitoring would need to continue for a longer time to attain more significant phase dispersal in these in vitro conditions. Besides the simple inertia, other forms of coupling independent of the action potentials may be sufficient to keep the SCN clock running ex vivo during the limited time of the recording [55, 56, 57]. Nevertheless, similar to the SCN tissue level, in TTX‐treated SCN explants, DEX increased relative amplitudes of the individual cellular rhythms more than vehicle.

## 5 Conclusion and Significance

These data suggest that the individual cells of the adult SCN are sensitive to the DEX treatment and respond by an increase in the amplitude similarly to cells of extra‐SCN brain clocks [19, 22], but the overall response is not that pronounced at the SCN tissue level due to the intercellular coupling. Previously, the causal relationship between disruptions in the SCN modulated GC rhythm and the etiology of neuropsychiatric disorders has been discussed (for review, see [58]). We propose an alternative hypothesis that the pathological states arising from chronodisruption may hypothetically result in impairment of the SCN clock robustness challenging its resilience to glucocorticoids with the consequences for the systemic output of the central clock.

## Author Contributions

**Martin Sládek:** experimental work, analysis, methodology, review of the manuscript. **Vendula Lužná:** experimental work, analysis, methodology, review of the manuscript. **Pavel Houdek:** experimental work, methodology, review of themanuscript. **Alena Sumová:** conceptualization, supervision, draft and final version of manuscript.

## Disclosure

The authors declare assistance of no AI technologies.

## Ethics Statement

Experiments are performed in accordance with a valid experimental project reference number AVCR 8271/2022 SOV II. The project is approved by the Animal Care and Use Committee of the Institute of Physiology of the Czech Academy of Sciences, as well as by the Resort Professional Commission of the CAS for Approval of Projects of Experiments on Animals. The animals are housed in facilities accredited by the Czech Ministry of Agriculture. Experiments are carried out under veterinary supervision, complying with Act no. 246/1992 Coll. and Decree no. 419/2012 Coll., implementing Directive 2010/63/EU of the European Parliament and of the Council regarding the protection of animals used for scientific purposes. The 3Rs principles are applied to the maximum extent possible.

## Conflicts of Interest

The authors declare no conflicts of interest.

## Data Availability

The datasets used and/or analyzed during the current study are available upon reasonable request from the corresponding author.
